# Depression-, Anxiety-, and Anger and Cognitive Functions: Findings From a Longitudinal Prospective Study

**DOI:** 10.3389/fpsyt.2021.665742

**Published:** 2021-08-06

**Authors:** Jutta Lindert, Kimberley C. Paul, Margie E. Lachman, Beate Ritz, Teresa E. Seeman

**Affiliations:** ^1^Department of Health and Social Work, University of Applied Sciences Emden/Leer, Emden, Germany; ^2^Women's Research Center at Brandeis University, Waltham, MA, United States; ^3^Department of Epidemiology, Jonathan and Karin Fielding School of Public Health, University of California, Los Angeles, Los Angeles, CA, United States; ^4^The Heller School for Social Policy and Management, Brandeis University, Waltham, MA, United States; ^5^Division of Geriatrics, David Geffen School of Medicine at UCLA, Los Angeles, CA, United States

**Keywords:** depression, anxiety, cognitive function, longitudinal, anger

## Abstract

**Background:** Determinants of changes in cognitive function during aging are not well-understood. We aimed to estimate the effects of depression-, anxiety- and anger symptoms on cognition and on cognition changes, especially on changes in episodic memory (EM) and executive functioning (EF).

**Methods:** We analyze data from the Mid-Life in the Midlife in the United States Biomarker study at two time points including *n* = 710 women, and *n* = 542 men (1996/1997) at the first assessment and *n* = 669 women, and *n* = 514 men at the second assessment (2013/2014). To assess cognition we used the Brief Test of Adult Cognition (BTACT). To measure depression-, anxiety- and anger symptoms we used the Mood and Anxiety Symptom Questionnaire (MASQ), the Center for Epidemiologic Studies Depression Scale (CES-D) and the State-Trait Anger Expression Inventory (STAXI). We used repeated models analyses to explore changes in cognition, and repeated measures linear mixed-effects models to investigate depression, anxiety and anger effects on cognition. All analyses were adjusted for potential confounders (cognition at baseline, age, education, income).

**Results:** At the first assessment, women had significantly better episodic memory functioning than men; men in the oldest age group had significant better executive functioning. At the second assessment, more education, and white ethnicity were associated with less negative changes on episodic memory and executive functioning. Depression- and anger symptoms were associated with declines in episodic memory among women; anxiety symptoms were associated with declines in episodic memory and executive functioning in both gender in men (EF: β: −0.02, (95% CI: −0.03, −0.01; EM: β −0.02 (−0.02, 95% CI: −0.03, −0.01) and in women (EF: β −0.01, 95% CI: −0.02, −0.0004; EM: β −0.013, 95% CI: −0.03, −0.001).

**Conclusions:** Depression-, anxiety- and anger symptoms were associated with changes in episodic memory and executive functioning. Further longitudinal studies are critical in populations in more countries to better understand the impact of depression, anxiety and anger symptoms on cognition changes.

## Introduction

Decline in cognitive function is a significant Public Health problem associated with disability, personal, and family suffering, institutionalization and increased risk for dementia. Cognitive function refers to the mental processes, which are critical for the conduct of everyday activities (1). Such mental processes include attention, short and long-term memory [episodic memory (EM)], reasoning and planning of tasks (executive function (EF) (2). Declines in cognitive function are possible with increasing age (3, 4). However, cognitive changes trajectories are heterogeneous and knowledge about modifiable risk factors is needed given the rising number of older people, worldwide (5) and the lack of effective treatments for cognitive function decline (6).

Studies suggest that a variety of socio-demographic and lifestyle factors are associated with cognitive function changes in the aging population such as gender and increasing age (7, 8), low childhood education (9, 10), smoking (11, 12), alcohol abuse (1) and social contact (13). The evidence for gender related cognitive function changes is mixed. In Northern Europe, women show higher EM levels than men, and in Southern Europe, women and men do not differ in cognitive function changes (14). Additionally to regional differences, gender differences in cognitive function tend to disappear among recently born cohorts (15, 16).

Additional to the socio-demographic and behavioral factors several aspects of emotions, especially depression and anxiety, have been implicated in risk of cognition decline (17–19). Studies suggest that ~20% of individuals aged 55+ experience depression- or anxiety symptoms (20, 21). Depression-, and anxiety symptoms are not only associated with physical health outcomes (22–24) such as cardiovascular diseases (25–27), and higher allostatic load (28) but with cognition decline (29–35).

Recent studies suggest that depression, anxiety and anger are associated with cognition decline and dementia risk. Depression has been recognized as potential risk factor for cognitive decline in a meta-analysis investigating risk and protective factors for cognition decline including 32 studies with 62 598 participants and a follow-up from 2 to 17 years, depression symptoms were a risk factor for dementia. However, studies showed substantial heterogeneity and main effects of depression on cognition decline was obvious in older cohorts of 80 years and older but less in younger individuals (36). Anxiety has been recognized as potential risk factor for cognitive in meta-analyses which found that people with anxiety disorders have a 29–45% greater risk of cognitive decline (37, 38). However, depression and anxiety are also part of the prodrome of dementia and associated with early stages of dementia. Therefore, reverse causation is possible whereby depression or anxiety symptoms result from changes in emotions long before cognition declines become recognizable (1). Additionally, there is some evidence, that more anger symptoms are associated with greater declines in cognitive function across long follow-ups (19, 39).

Finally, some studies suggest gender differences in cognitive function changes (40, 41). However, the gender differences in cognitive function vary across cohorts and regions (15, 16). To understand and support cognition changes in aging men and women a better understanding of the impact of depression-, anxiety- and anger symptoms on cognition changes is needed. This study aims to fill the gap in knowledge by investigating the association between depression-, anxiety- and anger symptoms and cognition changes. Specifically, our objective was to evaluate the association of depression-, anxiety- and anger symptoms on cognition changes, longitudinally.

## Methods

### Design

The study design is a longitudinal prospective cohort study.

### Participants

Participants were drawn from two waves of the Midlife in the US-Study (MIDUS-II (2004/2005) and MIDUS—III (2013/2014), i.e., the longitudinal follow-up of the original MIDUS-I cohort (1995/1996) (*n* = 7,108 participants, mean age 46 years, SD = 13). The original MIDUS-I cohort is a sample of non-institutionalized English speaking adults living in the 48 United States funded by the National Institute on Aging (42). The MIDUS participants were selected with a random digit dialing sampling procedure (43).

Our analytical sample consists of a subsample of MIDUS participants who provided information on anxiety-, depression-, and anger symptoms at MIDUS -II (the “Biomarker” subsample) and on cognition at MIDUS-II and MIDUS-III (44) (Supplemental Material Table 1: Flowchart of the analytical sample).

### Cognitive Function

Cognitive function was assessed with the Brief Test of Adult Cognition by Telephone (BTACT) (45) administered by telephone (46, 47). EM was assessed by the ability to correctly recall as many words read aloud from a 15-word list (word list immediate) and at the end of each BTACT interview after 20 min (word list delayed). Scoring is the number of unique words produced in 60 s or the number of unique words at the end of the interview. For the assessment of EF the following tests were used: Verbal fluency was tested in a test which asks participants to generate as many animals within 60 s as possible (score: number of animals produced in the 60 s); inductive reasoning was tested with the Number Series Test, which requires responding with the correct number in a series of numbers by inferring a pattern; processing speed is assessed with the 30-s-and-Counting-Task, which requires counting backwards from 100 within 30 s (score: total number of correct numbers reported); Attention switching/reaction time was assessed with the “stop” and “go” task. Following Lachman (46, 48), each score on EF was standardized and averaged to obtain a global EF score. The subtests were standardized using means and standard deviations from MIDUS-II. All cognitive assessment were administered by telephone interview.

### Depression-, Anxiety-, and Anger Symptoms

Participants reported on depression-, anxiety- or anger symptoms in the Mood and Anxiety Symptom Questionnaire (MASQ), the Center for Epidemiologic Studies Depression Scale (CES-D) and the State-Trait Anger Expression Inventory (STAXI) (Supplementary Material Table 1). The MASQ was designed to assess symptoms of distress, depression, and anxiety (49) on two subscales. The distress scale contains mixed anxiety and somatic symptoms (11 items) and the anxiety scale contains anxiety symptoms (17 items) (50). The Center for Epidemiological Studies Depression (CES-D**)** Scale is a self-report scale designed to measure depression symptoms in the (51) with 20-items on a four-point Likert scale. We measured furthermore anger using the STAXI. The STAXI assesses anger-in and anger out with each eight items on a four-point Likert scale; and anger control with four items on a seven-point scale (52, 53). For each scale, we calculated the mean score of all items allowing for up to one missing item per respondent.

### Co-variates

Socio-demographic- and economic variables were measured at baseline with the MIDUS questionnaire. We assessed chronological age (in categories), gender (female, male), race/ethnicity (White, Latino, other), marital status (married, not married), educational level (high school or less, some college or more), working status (employed, unemployed, retired/other not working), and income. Income was measured adjusted for household size to generate a measure relative to the Federal Poverty Level (FPL); i.e., a measure of annual cash income issued every year by the Department of Health and Human Services in the US (for 2018: $12,140 for individuals). To categorize FPL for this study, we calculated FPL-tertiles for the respective study years (tertile 1: <305 FPL, tertile 2: 306–564 FPL, tertile 3: >565%).

### Data Analyses

First, we calculated cross-sectional descriptive statistics by gender and age (including standardized means for EF and EM). Second, we assessed the correlations between emotional items with Pearson's R. Next, we assessed cross-sectional associations between depression, anxiety and anger and EM and EF age, gender, marital status, race/ethnicity, educational level, and income cross-sectionally using linear regression analyses. To investigate changes in EM and EF from time-point one to time-point two, we estimated linear mixed-effects repeated measures models. We conducted longitudinal hierarchical block-wise linear repeated measures regression analysis. In a block-wise manner, we adjusted for baseline cognition and performed blockwise regression analyses. First we regressed the EM and EF scores on sociodemographic variables (gender, marital status, race/ethnicity, and education), forming residuals. Then we regressed these residuals on working status and household income, forming a second set of residuals without the variance explained by block one and two. Third mental condition symptoms (distress, depression, and anger), were entered into the regression model. Missing data in the sample were handled based on a missing-at-random assumption. To investigate the robustness of our findings we conducted sensitivity analyses in which we excluded one of two siblings who were enrolled in the study or non-white participants. All analyses were conducted in SASv9.4 (SAS Institute Inc.).

## Results

We included *n* = 1,252 (*n* = 542 (43.3% male) at MIDUS II, and *n* = 1,183 [*n* = 514 male (43.5% male)] at MIDUS-III (Supplementary Material Table 1). The majority of participants were white (*N* = 821, 87%) with a mean age of 54.9 (S.D. = 11.8) at the first time point and 63.4 (S.D. = 11.1) at the second time point, and slightly more were women (56.5%). A majority of the male participants reported some college education or more (75.9%). Female participants were less often married than men were. Mean levels of household income were higher in men than in women.

To describe cognition and depression-, anxiety-, and anger symptoms in the analytical sample, we analyzed means and standard deviations for EM and EF scores in men and women at the two exams (Table 1). Consistent with other findings on the MIDUS sample (48), in our analytical sample women had higher EM mean scores across all age groups at both exams compared to men, while men had higher EF scores across all age groups at both exams (Table 1). Among participants, mean EM and EF declined with increasing age. Women exhibited higher levels of anxiety and depression than men at the second exam (Table 1).

**Table 1:** Means and standard deviations for EM and EF scores by gender at the two exams.

	**First exam**							**Second exam**						
	**Men**			**Women**				**Men**			**Women**			
**Age group**	***n***	**Mean, SD**	**Range**	***n***	**Mean, SD**	**Range**	***p-*value**	***n***	**Mean, SD**	**Range**	***n***	**Mean, SD**	**Range**	***p*-value**
All	493	−0.25, 0.89	−2.10–2.57	652	0.25, 0.89	−2.50–3.23	<0.0001	414	−0.34, 0.84	−2.10–2.63	552	0.26, 1.04	−2.50–3.64	<0.0001
40–49	132	−0.01, 0.72	−1.88–2.41	169	0.45, 0.79	−1.47–2.44	<0.0001	33	0.10, 0.89	−1.44–2.03	60	0.60, 1.09	−1.47–3.42	0.028
50–59	147	−0.19, 0.71	−1.88–1.56	205	0.32, 0.90	−1.66–3.23	<0.0001	105	0.05, 0.85	−1.88–2.63	132	0.55, 1.00	−1.44–3.64	<0.0001
60–69	105	−0.50, 0.74	−2.10–2.57	137	0.12, 0.74	−1.66–2.44	<0.0001	124	−0.32, 0.72	−2.10–2.60	145	0.40, 0.91	−1.88–2.79	<0.0001
≥70	73	−0.69, 0.73	−2.10–0.96	72	−0.33, 0.56	−1.66–2.82	0.006	119	−0.75, 0.69	−1.88–1.56	142	−0.13, 1.04	−2.50–2.65	<0.0001
All	495	0.13, 0.65	−2.58–2.01	654	−0.001, 0.65	−3.07–1.93	0.001	415	−0.01, 0.69	−2.22–1.97	553	−0.19, 0.74	−3.15–1.71	0.002
40–49	132	0.40, 0.64	−0.99–2.01	169	0.22, 0.62	−2.01–1.51	0.018	33	0.34, 0.66	−1.07–1.49	60	0.23, 0.63	−1.06–1.71	0.445
50–59	147	0.17, 0.58	−1.38–1.50	206	0.05, 0.61	−1.77–1.56	0.051	105	0.38, 0.67	−1.41–1.97	132	0.18, 0.59	−1.74–1.41	0.016
60–69	105	−0.06, 0.55	−1.16–1.33	138	−0.19, 0.63	−3.07–1.30	0.088	124	0.04, 0.55	−1.26–1.35	145	−0.05, 0.64	−1.66–1.58	0.194
≥70	73	−0.29, 0.63	−2.58–1.30	72	−0.50, 0.51	−2.02–0.64	0.025	120	−0.34, 0.55	−1.58–0.96	143	−0.57, 0.65	−3.15–1.36	0.002

Correlations among the variables are shown in Supplementary Material Table 2. Measures for depression and anxiety symptoms correlated strongly.

An initial set of analyses examined within person changes in EM and EF. For EM, significant within-person changes between exams were not observed until participants reached 70 years of age (women) and 60 years of age (men) (Supplementary Material Table 3).

### Depression-, Anxiety-, and Anger Symptoms and Cognition

We observed different associations between depression-, anxiety-, and anger symptoms with EM and EF (Table 2). At the first exam, higher age, being of race/ethnicity other than white, lower education, being unemployed and lower income was associated with worse EM and EF scores. More anxiety symptoms were associated with worse EM and EF, while less anger symptoms was associated with better EF. In women, higher age, being of race/ethnicity other than white and lower education was associated with worse EM and EF. However, not being married and low income were associated with worse EF only. Additionally, depression- and anxiety symptoms but not anger symptoms were associated with worse EM and EF.

**Table 2:** Associations of socio-demographic, work related, and mental conditions (depressive-, anxiety-, and anger symptoms) and executive function and episodic memory by gender in cross-sectional analyses.

	**Men**				**Women**			
	**Executive function**		**Episodic memory**		**Executive function**		**Episodic memory**	
	**(***n=*****558)****		**(***n=*****556)****		**(***n=*****558)****		(***n=*** **556)**	
	**β**	**95% CI**	**β**	**95% CI**	**β**	**95% CI**	**β**	**95% CI**
**Socio-Demographics**								
Baseline age	−0.02	−0.02, −0.01^*^	−0.02	−0.02, −0.01^*^	−0.02	−0.02 −0.02^*^	−0.02	0.03, −0.02^*^
**Ethnicity (Reference:White)**								
Latino	−0.42	−0.73, −0.11^*^	0.02	−0.46, −0.01^*^	−0.25	−0.47, −0.03^*^	−0.06	−0.41, 0.28
Other	−0.09	−0.33, – 0.12^*^	−0.17	−0.69, −0.29^*^	−0.45	−0.65, −0.25^*^	−0.38	−0.70, −0.07^*^
**Education (Reference: Less Than College Education)**								
Some college	0.30	0.17, 0.43^*^	0.27	−0.18, 0.01	0.33	0.25, 0.43^*^	0.25	0.11, 0.39^*^
**Marital status (reference: married)**								
Not married	−0.12	−0.25, 0.01	0.02	−0.18, 0.01	−0.12	−0.21, −0.02	0.02	−0.13, 0.16
**Economic Variables**								
**Working status (reference: working)**								
Unemployed	−0.55	−0.92, −0.17^*^	−0.73	−0.22, 0.05	−0.14	−0.27, −0.01^*^	0.08	−0.13, 0.29
Retired/other	−0.09	−0.24, 0.07	−0.17	−0.30, −0.05^*^	−0.19	−0.31, −0.07^*^	−0.13	−0.32, 0.06
**Household income (reference: tertile 3)**								
Tertile 1	−0.12	−0.26, 0.02	−0.11	−0.35, −0.11^*^	−0.22	−0.34, −0.10^*^	−0.27	−0.46, 0.09
Tertile 2	−0.02	−0.14, 0.11	−0.17	−0.21, 0.02	0.08	−0.19, 0.04	−0.14	−0.31, 0.04
**Mental Health**								
Depression 1^*^	−0.004	−0.01, 0.004	−0.01	−0.02, −0.01^*^	−0.01	−0.02, −0.01^*^	−0.01	−0.02, 0.001
Distress	0.003	−0.01, 0.01	0.000	−0.02, −0.003^*^	−0.01	−0.02, −0.01^*^	−0.01	−0.02, −0.01^*^
Depression 2^**^	0.01	−0.01, 0.02	0.002	−0.02, −0.003^*^	−0.01	−0.02, −0.003^*^	−0.01	−0.02, −0.001^*^
Anxiety^*^	−0.01	−0.03, −0.001^*^	−0.02	−0.02, −0.003^*^	−0.02	−0.02, −0.006^*^	−0.01	−0.02, 0.003
Anger^***^								
**State anger (reference: no state anger)**
Anger—in	0.01	−0.003, 0.03	0.01	−0.02, 0.003	−0.01	−0.02, −0.01^*^	−0.01	−0.03, 0.01
Anger—out	0.004	−0.01, 0.02	0.01	−0.02, 0.02	−0.002	−0.02, 0.01	−0.01	−0.02, −0.001^*^
**Trait Anger/Reference: No Trait Anger)**								
Trait anger	0.006	−0.004, 0.02	0.01	−0.01, 0.01	−0.003	−0.01, 0.01	0.000	−0.01, 0.01

* *Assessed with the MASQ:*

** *Assessed with the CESD;*

*** *Assessed with the STAXI*.

In repeated measures analyses, we assessed the impact of a variety of socio-demographic,- and work related mental conditions related factors on EM and EF in block-wise analyses (Table 3). In men, lower levels in EF at follow-up were associated with higher age, race/ethnicity other than white and being unemployed, and lower levels in EM were associated with being unemployed and lower household income. In women, lower levels in EF were associated with higher age, and race/ethnicity other than white, not being married, being retired and lower household income. A decline in EM was associated with race/ethnicity other than white and lower education.

**Table 3:** Effects of socio-demographic and -economic variables on executive function (EF) and episodic memory (EM) 10 years after baseline, adjusted for baseline age, time, socio-demographic and—economic variables.

	**Men**				**Women**			
	**Executive function**		**Episodic memory**		**Executive function**		**Episodic memory**	
	**β**	**95% CI**	**β**	**95% CI**	**β**	**95% CI**	**β**	**95% CI**
	***N=*** **365**		***N=*** **364**		***N=*** **465**		***N=*** **463**	
**Variable**								
Follow-up time	−0.02	−0.03, −0.01	−0.02	−0.03, 0.004	−0.03	−0.03, −0.02	−0.004	−0.02, 0.01
Baseline age	−0.02	−0.03, −0.02	−0.03	−0.03, −0.02	−0.03	−0.03, −0.02	−0.02	−0.03, −0.02
**Socio-Demographics**								
**Ethnicity (reference: white)**								
Latino	−0.77	−0.63, −0.12	−0.04	−0.25, 0.28	−0.19	−0.37, −0.001	−0.09	−0.39, 0.20
Other	−0.23	−0.43, −0.04	−0.13	−0.36, 0.11	−0.47	−0.64, 0.29	−0.33	−0.60, −0.05
**Education**								
Some college	0.21	0.11, 0.31	0.20	0.06, 0.34	0.36	0.28, 0.43	0.27	0.15, 0.38
**Marital status (reference: married)**								
Not married	−0.09	−0.20, 0.03	0.07	−0.07, 0.20	−0.10	−0.18, −0.03	0.01	−0.11, 0.12
**Economic Variables**								
	*n =* 357	*n =* 356	*n =* 459	*n =* 459
**Working status (reference: employed)**								
Unemployed	−0.54	−0.87, 0.20	−0.45	−0.86, −0.04	−0.09	−0.21, 0.02	0.11	−0.07, 0.28
Retired/other	−0.08	−0.20, 0.04	−0.08	−0.22, 0.66	−0.12	−0.20, −0.04	−0.01	−0.15, 0.12
**Household income (reference: tertile 3)**								
Tertile 1^+^	−0.08	−0.19, 0.04	−0.11	−0.25, 0.03	−0.16	−0.24, 0.08	−0.17	−0.30, −0.03
Tertile 2^++^	−0.002	−0.10, 0.10	−0.14	−0.25, −0.02	−0.07	−0.15, 0.01	−0.06	−0.20, 0.08

+ *Tertile 1 <305%FPL,*

++ *Tertile 2: 306–564%FPL*.

Finally, we examined whether depression-, anxiety- or anger symptoms influence EM and EF change over a decade, accounting for s *socio-demographic,- and work related* covariates (Table 4). We found that in both, in men and women, more anxiety symptoms were statistically significantly associated with worse EM and EF. Additionally, in women higher depression symptoms were significantly associated with worse EM and EF. In men, more trait anger symptoms were associated with better EM. In contrast, in women, more sate anger symptoms contributed to worse EM.

**Table 4:** Effects of mental health (depressive-, anxiety-, or anger symptoms) on executive function and episodic memory 10 years after baseline on men and women, adjusted for baseline age, time, socio-demographic and—economic variables United States, 2016.

	**Men**				**Women**			
	**Executive function**		**Episodic memory**		**Executive function**		**Episodic memory**	
**Variable**	**β**	**95% CI**	**β**	**95% CI**	**β**	**95% CI**	**β**	**95% CI**
	***N=*** **365**		***N=*** **364**		***N=*** **465**		***N=*** **463**	
**Mental conditions**								
**Distress (reference: no distress)**								
Distress^*^	0.001	−0.01, 0.01	−0.003	−0.01, 0.01	−0.008	−0.01, −0.002^*^	−0.012	−0.02, −0.003
**Depression (referencce: no depression)**								
Depression 1^*^	0.001	−0.01, 0.01	−0.003	−0.01, 0.01	−0.008	−0.01, −0.002	−0.012	−0.02, −0.002
**Anxiety (reference: no anxiety)**								
Anxiety^*^	−0.02	−0.03, −0.01	−0.02	−0.03, −0.01	−0.01	−0.02, −0.004	−0.013	–0.03, −0.001
**Depression 2 (reference: no depression 2)**								
Depression 2^**^	−0.002	−0.01, 0.01	−0.006	−0.01, 0.002	−0.008	−0.01, −0.004	−0.01	−0.02, 0.002
**Anger**								
State anger (Anger-in)^***^	0.007	−0.02, 0.001	0.002	−0.01, 0.02	0.007	−0.02, 0.001	−0.006	−0.02, −0.01
Trait anger^***^	0.004	−0.004, 0.01	0.01	0.002, 0.02	0.004	−0.002, 0.01	0.001	−0.01, 0.01

* *Assessed with the MASQ:*

** *Assessed with the CESD;*

*** *Assessed with the STAXI*.

### Sensitivity Analyses

We conducted two sensitivity analyses. First, to investigate whether the twins which are included in the sample influenced results, we analyzed data excluding one twin from each pair from all analyses. Results were robust and any differences are likely related to the reduction of statistical power in the smaller sample. Second, to investigate whether race/ethnicity (white, Latino, and Latina) had an impact on the results, we analyzed participants of white race/ethnicity vs. race/ethnicity other than white and found no more than minor changes in effect estimates.

## Discussion

Using data of a large longitudinal sample of middle aged and older individuals, the present study examined the long-term associations between depression-, anxiety-, and anger symptoms and EM and EF. Consistently, our analyses suggest that depression-, anxiety-, and anger symptoms are related to cognition. In repeated measures analysis, higher anxiety was associated with lower levels of EM and EF. Analyzing the data by female and male gender, we found that in women, more depression symptoms were associated with worse EM; and that in men, more anger symptoms were associated with better EM.

Overall, we found age-related EM and EF changes which have been found previously in studies analyzing the MIDUS data (48). We observed decreases in cognition after age 70. Prior studies with other samples suggested age-related changes after age 60 (54). The difference of a decade might be related to worldwide increases in cognitive performances favoring later born cohorts in many population groups. Cognitive performance increases have been found in more recent birth cohorts and have been attributed to changes in living conditions, family size, health, and better education (15, 16).

The pattern for cognition confirms the results of previous studies with data from respondents ages 50–93 years from other longitudinal surveys (14), suggesting that women outperform same age men on EM (55). Studies conducted in non-Western societies, such as China and India, however, have reported a disadvantage for older women compared with men in the same age group in measures of EM and word fluency.

### Depression and Cognition

Adjusting for all covariates, more depression symptoms were associated with worse EM and EF in women. The findings are in line with previous research which suggested that individuals with mood disorders have worse cognition (56), and that positive emotions help maintain cognitive function (57, 58). Furthermore, in women, depression symptoms are associated with worse EM and EF. Depression symptoms may trigger hypercortisolemia (59, 60), increase autonomic responses associated with cardiovascular disease (61, 62), contribute to inflammation (63), and herewith to neurotoxicity. These processes may accelerate cognitive decline during aging (64).

### Anxiety and Cognition

We show that higher levels of anxiety symptoms are associated with lower levels of EM and EF in men and women. An explanation for the adverse effects of increased anxiety on decreases in EF and EM is given by the attentional control theory (65, 66). Attentional control theory predicts that anxiety impairs various functions (66, 67), specifically, the inhibition, shifting and updating functions and herewith reduces resources for EM and EF (68, 69). A further explanation might be that anxiety is associated with anxiety induced hypothalamic-pituitary-adrenal (HPA) over-activation, and chronic changes in cortisol levels, which may lead to dysregulation of the immune system, damage of the hippocampus and subsequent changes in cognition.

### Anger and Cognition

More anger symptoms are associated with better EM and worse EF. It has been suggested that anger symptoms are associated with reductions in cortisol (70, 71). The associations were small, however, they are important from a Public Health perspective as small changes in a huge population are of huge importance from a Public Health Point of view.

Strengths of our study include the longitudinal design, well-validated measures and the 10 years of follow-up. Additional strengths are that we were able to investigate these effects in a large population-based sample with information on multiple cognitive domains as well as detailed confounding variables. The study used measures that are useful in community-based studies of non-clinical populations. An additional strength of this study is that we analyzed data separately for men and women. Assessment of depression-, anxiety- and anger symptoms was based on self-report and therefore some misclassification is likely. Any such misclassification, however, is expected to bias our results toward the null. The findings concerning non-white populations are based on a small sample size. It might be that the results of the findings are limited due to this sample size. Additionally, this study is an observational study, which does not test mental health symptoms, EM, and EF under standardized conditions. However, sensitivity analyses analyzing a “white only” sample yielded the same results, suggesting an association of increased depression and anxiety symptoms with decreased EM and EF. Attrition is a concern when carrying out longitudinal research. It might be that individuals in poorer health tend not to participate in longitudinal cohort studies. We can report changes between two time points but not trajectories based on multiple assessments. The MIDUS sample underrepresents those with lower levels of education. A further limitation might be that the sample we used is based on the Biomarker study only, which is a subsample of the MIDUS II study. However, these respondents were quite similar to the sample, which they were recruited from (44). The differences between the baseline sample and the follow up sample might have introduced some selection bias if loss was differential according to exposure and outcome. Specifically, as individuals with more severe cognitive limitations tend to participate less in studies at follow-up and those with certain mood disorders specifically depression also are more likely lost, we may have underestimated the impact of such emotions on cognitive outcomes.

The findings of this study suggest a long-term association between depression-, anxiety- and anger symptoms in midlife and EM and EF in later life. EM and EF in aging populations can be improved by reducing depression-, anxiety- and anger symptoms. In the future differences between men in women in the effect of the different emotional factors on cognitive changes over time should be assessed. Our findings have important implications for preventive interventions. Although some aspects of early life cannot be modified such as early life education, later lie conditions such as depression-, anxiety-, or anger symptoms might be modifiable. Interventions to reduce depression-, anxiety-, and anger symptoms in midlife may benefit EM and EF in later life.

## Data Availability Statement

The raw data supporting the conclusions of this article will be made available from the authors, without undue reservation.

## Ethics Statement

Data collection for all phases of the MIDUS studies was approved by Institutional Review Boards and all participants provided informed consent.

## Author Contributions

JL, BR, MEL, and TES designed this study. TES and MEL collected the data for the MIDUS studies. JL, BR, and KCP developed the analysis strategy. KCP analyzed the data. JL wrote the manuscript. All authors commented on the manuscript and approved the manuscript.

## Conflict of Interest

The authors declare that the research was conducted in the absence of any commercial or financial relationships that could be construed as a potential conflict of interest.

## Publisher's Note

All claims expressed in this article are solely those of the authors and do not necessarily represent those of their affiliated organizations, or those of the publisher, the editors and the reviewers. Any product that may be evaluated in this article, or claim that may be made by its manufacturer, is not guaranteed or endorsed by the publisher.
