# Supplementary material for: Depression-, Anxiety-, and Anger and Cognitive Functions: Findings From a Longitudinal Prospective Study
Source: Front Psychiatry. 2021 Aug 6;12:665742. doi: 10.3389/fpsyt.2021.665742 (PMC8377351; doi:10.3389/fpsyt.2021.665742)
Supplement: Supplementary file 1 [file Data_Sheet_1.docx]

**Supplemental material**

**Flow chart of the analytical sample based on the MIDUS subsample the “Biomarker” project (exam 1, baseline analytical sample; exam 2, follow-up analytical sample) of the MIDUS study**

**Table 1.** Depressive-, anxiety- or anger symptoms measures (Characteristics, mean score, range, IQR, coefficient Variation) used at baseline

| **Affect measure** | **Characteristic**  **assessed** | **Mean score**  **(SD+)** | **Range** | **IQR** | **Coefficient Variation** |
| --- | --- | --- | --- | --- | --- |
| MASQ-distress scale | Nonspecific general distress or negative affect symptoms | 18.6 (6.7) | 12-60 | 7.0 | 36.1 |
| MASQ depression | Symptoms of anhedonic depression | 16.70 (4.8) | 11-47 | 6.0 | 28.9 |
| MASQ anxiety | Symptoms of anxious arousal | 22.0 (5.4) | 17-70 | 6.0 | 24.9 |
| CES-D depression | Depression proneness | 8.5 (8.0) | 0-54 | 9.6 | 94.8 |
| STAXI trait anger | Anger proneness over time | 23.8 (5.4) | 15-54 | 7.0 | 22.6 |
| STAXI anger expression-in | Tendency to suppress anger feelings | 14.6 (4.1) | 8-31 | 5.0 | 28.3 |
| STAXI anger-expression-out | Tendency to express anger towards other persons or objects | 12.9 (3.3) | 8-29 | 5.0 | 25.4 |
| STAXI anger-control-out | Control of anger feelings by preventing the expression of anger toward other persons or objects | 10.0 (2.3) | 4-14 | 4.0 | 22.7 |
| STAXI anger adjustment ( | Managing anger feelings | 2.1 (0.7) | 1-4 | 1.0 | 32.4 |

Higher scores on each measure denote more of the characteristic.

*SD, standard deviation; CES-D, Center for Epidemiologic Studies Depression; STAXI: State-Trait Anger Expression Inventory

**Table 2. Baseline (n=1252) and 10 years’ follow-up (n=1183) characteristics of study sample by gender**

|  | **Wave 1 (MIDUS – II)** | | | | | | **Wave 2 (MIDUS – III)** | | | | | |
| --- | --- | --- | --- | --- | --- | --- | --- | --- | --- | --- | --- | --- |
|  | **Men** |  | **Women** |  | **Total** |  | **Men** |  | **Women** |  | **Total** |  |
|  | n | % | n | % | n | % | n | % | n | % | n | % |
| **Participants** | 542 | 43.3 | 710 | 56.7 | 1252 | 100 | 514 | 43.5 | 669 | 56.5 | 1183 | 100 |
| **Age** |  |  |  |  |  |  |  |  |  |  |  |  |
| mean/SD | 55.5 (12.0) | | 54.4 (11.5) | | 54.9 (11.8) | | 63. 8 (11.2) | | 63.4 (11.1) | | 63.4 (11.1) | |
| <40 | 39 | 7.7 | 73 | 10.9 | 112 | 9.5 | 0 |  | 0 |  | 0 |  |
| 40-49 | 139 | 27.4 | 176 | 26.8 | 315 | 26.7 | 39 | 9.3 | 68 | 13,0 | 107 | 11.3 |
| 50-59 | 149 | 29.4 | 209 | 30.1 | 358 |  | 117 | 27.9 | 146 | 27.9 | 263 | 27.9 |
| 60-69 | 107 | 21.1 | 140 | 21.5 | 247 |  | 136 | 32.4 | 157 | 30.0 | 293 | 31.0 |
| <69 | 73 | 14.4 | 73 | 11.5 | 146 |  | 128 | 30.1 | 153 | 29.2 | 281 | 29.8 |
| Missing | 35 |  | 39 |  | 74 |  |  |  |  |  | 239 |  |
| **Race/Ethnicity** | |  |  |  |  |  |  |  |  |  |  |  |
| White | 438 | 9.2 | 521 | 90.9 | 959 | 91.5 | 369 | 88.5 | 452 | 86.9 | 821 | 87.6 |
| Hispanic | 14 | 2.9 | 23 | 4.0 | 37 | 3.5 | 13 | 3.1 | 22 | 4.2 | 35 | 3.7 |
| Other | 23 | 4.8 | 29 | 5.1 | 52 | 5.0 | 35 | 8.4 | 46 | 8.9 | 81 | 8.6 |
| Missing | 67 |  | 137 |  | 204 |  | 97 |  |  |  | 246 |  |
| **Marital status** | |  |  |  |  |  |  |  |  |  |  |  |
| Married | 379 | 79.8 | 381 | 66.2* | 760 | 72.31 | 381 | 66.2 | 316 | 75.2 | 632 | 66.9 |
| Separated /divorced/  widowed | 52 | 11.0 | 154 | 26.7* | 206 | 19.6 | 321 |  | 327 |  | 243 | 25.7 |
| Never married | 44 | 9.3 | 41 | 7.1 | 85 | 8.1 | 34 | 8.1 | 35 | 6.7 | 69 | 7.3 |
| Missing | 67 |  | 134 |  | 201 |  |  |  |  |  | 239 |  |
| **Education** |  |  |  |  |  |  |  |  |  |  |  |  |
| High school or less | 105 | 22.1 | 183 | 31.8* | 288 | 27.4 | 104 | 22.0 | 183 | 31.8 | 287 | 27.4 |
| Some college or higher | 371 | 77.9 | 393 | 68.2* | 764 | 72.6 | 369 | 78.0 | 392 | 68.2 | 761 | 72.6 |
| Missing | 66 |  | 134 |  | 202 |  | 41 |  | 94 |  | 135 |  |
| **Working status** | |  |  |  |  |  |  |  |  |  |  |  |
| Employed/  students | 354 | 74.4 | 365 | 63.6* | 719 | 68.5 | 251 | 70.3 | 262 | 60.9 | 513 | 65.2 |
| Retired  /other | 112 | 23.5 | 136 | 23.17 | 248 | 21.9 | 97 | 27.2 | 116 | 27.0 | 213 | 27.1 |
| Unem-ployed | 10 | 2.1 | 73 | 12.7 | 83 | 7.9 | 5 | 1.4 | 41 | 9.5 | 46 | 5.8 |
| Other | 3 | 0.6 | 15 | 2.6 | 18 | 1.7 | 4 | 1.1 | 11 | 2.6 | 15 | 1.9 |
| Missing | 66 |  | 136 |  | 202 |  | 157 |  |  |  | 396 |  |
| **Income** |  |  |  |  |  |  |  |  |  |  |  |  |
| % FPL mean | 535, 10 | 395, 80 | 480, 90 | 381, 00 | 50, 29 | 388,48 | 51, 00 | 430, 90 | 440, 90 | 396, 30* | 474, 47 | 413, 40 |
| Tertile 1: <305% FPL | 132 | 28.5 | 209 | 36.9 | 341 | 33.1 | 99 | 27.4 | 174 | 37.9* | 273 | 33.3 |
| Tertile 2: 306-564% FPL | 165 | 35.6 | 181 | 31.9 | 346 | 33.6 | 130 | 35.9 | 144 | 31.4 | 274 | 33.4 |
| Tertile 3:  >565% FPL | 167 | 36.0 | 177 | 31.2 | 344 | 33.3 | 133 | 36.7 | 141 | 30.7* | 274 | 33.4 |
| Missing | 78 | - | 143 | - | 221 | - | 152 | - | 210 | - | 362 | - |
| **Symptom mean (SD) and range** | | | | |  |  |  |  |  |  |  |  |
| Anxiety | 16.1 (4.5) | 11-39 | 17.1* (5.1) | 11-42 | 16.7 (4.9) | 11-42 Not applicable. scales not conducted in exam 2 | | | | | | |
| CESD | 8.3 (7.8) | 0-54 | 9.1 (8.4) | 0-49 | 8.7 (8.2) | 0-54  8-31  8-29  1-4 | | | | | | |
| Anger in | 14.8 (4.1) | 8-30 | 14.5 (4.2) | 8-31 | 14.6 (4.2) |  |  |  |  |  |  |  |
| Anger out | 12.8 (3.2) | 8-28 | 13.0 (3.4) | 8-29 | 12.9 (3.3) |  |  |  |  |  |  |  |
| Anger - adjustment | 2.2 (0.7) | 1-4 | 2.0 (0.7) | 1-4 | 9.9 (2.3) |  |  |  |  |  |  |  |
| Anger-control | 10.1 (2.2) | 4-13 | 9.8 (2.3) | 4-14 | 9.9 (2.3) | 4-14 | | | | | | |
| Trait anger | 23.8 (5.4) | 15-54 | 23.9 (5.6) | 15-47 | 23.8 (5.5) | 15-54 | | | | | | |

*Federal Poverty Guidelines (reference), **p<0.05 comparing men and women, t-test or chi square

| **Supplemental Material. Table 3. Correlations between study variables at baseline** | | | | | | | | | | | | | | | | |
| --- | --- | --- | --- | --- | --- | --- | --- | --- | --- | --- | --- | --- | --- | --- | --- | --- |
|  | | 1 | 2 | | 3 | 4 | 5 | 6 | 7 | 8 | 9 | 10 | 11 | 12 | 13 | 14 |
| Gender | | 1.00 | -0.04 | | 0.28 | -0.10 | 0.07 | 0.10 | 0.11 | 0.05 | -0.04 | 0.02 | -0.08 | 0.01 | 0.29 | -0.11 |
| p-value |  | - | 0.1479 | | <.0001 | 0.0007 | 0.0093 | 0.0003 | <.0001 | 0.0958 | 0.1495 | 0.5548 | 0.0055 | 0.7067 | <.0001 | 0.0003 |
| 2. Age |  |  | 1.00 | | -0.28 | -0.34 | -0.17 | -0.15 | 0.00 | -0.14 | -0.25 | -0.21 | 0.07 | -0.12 | -0.30 | -0.39 |
| p-value |  |  | - | | <.0001 | <.0001 | <.0001 | <.0001 | 0.946 | <.0001 | <.0001 | <.0001 | 0.0137 | <.0001 | <.0001 | <.0001 |
| 3. Episodic memory |  |  |  | | 1.00 | 0.39 | 0.00 | 0.05 | 0.06 | -0.07 | 0.04 | 0.07 | 0.02 | 0.04 | 0.53 | 0.31 |
| p-value |  |  |  | | - | <.0001 | 0.9665 | 0.1216 | 0.0489 | 0.0165 | 0.147 | 0.022 | 0.6043 | <.0001 | <.0001 | <.0001 |
| 4. Exec-utive func-tioning |  |  |  | |  | 1.00 | -0.04 | -0.02 | -0.16 | -0.15 | 0.08 | 0.06 | 0.10 | 0.04 | 0.78 | 0.78 |
| p-value |  |  |  |  | | - | 0.2992 | 0.4585 | <.0001 | <.0001 | 0.0072 | 0.0373 | 0.008 | 0.2045 | <.0001 | <.0001 |
| 5. Distress |  |  |  |  | |  | 1.00 | 0.70 | 0.55 | 0.82 | 0.46 | 0.25 | -0.19 | 0.45 | -0.04 | -0.05 |
| p-value |  |  |  |  | |  | - | <.0001 | <.0001 | <.0001 | <.0001 | <.0001 | <.0001 | <.0001 | 0.2265 | 0.0882 |
| 6. De-pression I* |  | |  |  | |  |  | 1.00 | 0.67 | 0.64 | 0.41 | 0.27 | -0.15 | 0.47 | -0.02 | -0.06 |
| p-value |  |  |  |  | |  |  | - | <.0001 | <.0001 | <.0001 | <.0001 | <.0001 | 0.0392 | 0.5411 | 0.0454 |
| 7. Anxiety |  | |  |  | |  |  |  | 1.00 | 0.54 | 0.28 | 0.23 | -0.15 | 0.37 | -0.10 | -0.20 |
| p-value |  |  |  |  | |  |  |  | - | <.0001 | <.0001 | <.001 | <.0001 | <.0001 | <.0001 | <.0001 |
| 8. De-pression II* |  | |  |  | |  |  |  |  | 1.00 | 0.46 | 0.22 | -0.23 | 0.45 | -0.09 | -0.14 |
| p-value |  |  |  |  | |  |  |  |  | - | <.0001 | <.0001 | <.0001 | <.0001 | <.0001 | <.0001 |
| 9. Anger-in |  | |  |  | |  |  |  |  |  | 1.00 | 0.26 | -0.14 | 0.53 | 0.04 | 0.08 |
| p-value |  |  |  |  | |  |  |  |  |  | - | <..0001 | <..0001 | <..0001 | 0.1978 | 0.0156 |
| 10. Anger-out |  | |  |  | |  |  |  |  |  |  | 1.00 | -0.27 | 0.57 | 0.08 | 0.07 |
| p-value |  |  |  |  | |  |  |  |  |  |  | - | <.0001 | <.0001 | 0.0098 | 0.0186 |
| 11. Anger-control |  | |  |  | |  |  |  |  |  |  |  | 1.00 | -0.24 | 0.01 | 0.06 |
| p-value |  |  |  |  | |  |  |  |  |  |  |  | - | <.0001 | 0.811 | 0.061 |
| 12. Trait Anger |  | |  |  | |  |  |  |  |  |  |  |  | 1.00 | 0.05 | 0.04 |
| p-value |  |  |  |  | |  |  |  |  |  |  |  |  | - | 0.0946 | 0.2069 |
| 13. Epi-sodic memory |  |  |  |  | |  |  |  |  |  |  |  |  |  | 0.62 | 0.90 |
| p-value |  |  |  |  | |  |  |  |  |  |  |  |  |  | <.0001 | <.0001 |
| 14. Execu-tive func-tioning |  |  |  |  | |  |  |  |  |  |  |  |  |  | - | 1.00 |
| p-value |  |  |  |  | |  |  |  |  |  |  |  |  |  |  | - |

| **Supplemental Material. Table 4. Within person changes in episodic memory and executive functioning from baseline to follow - up, by age groups** | | | | | | | | | |  |
| --- | --- | --- | --- | --- | --- | --- | --- | --- | --- | --- |
|  | n | **Total Population** | | | **Men** | | | **Women** | |  |
|  |  | Within person mean difference (SD) | p-value | n | Within person mean difference (SD) | p-value | n | Within person mean difference (SD) | p-value |  |
| **Episodic Memory** | | | | | | | | | |  |
| All | 990 | -0.088 (0.91) | 0.0034 | 397 | -0.153 (0.83) | 0.000 | 593 | -0.039 (0.97) | 0.347 |  |
| 40-49 | 88 | -0.033 (0.92) | 0.7345 | 31 | -0.187 (1.02) | 0.317 | 57 | 0.050 (0.85) | 0.657 |  |
| 50-59 | 227 | 0.043 (0.97) | 0.5020 | 100 | -0.008 (0.91) | 0.926 | 127 | 0.084 (1.02) | 0.353 |  |
| 60-69 | 260 | -0.096 (0.86) | 0.0732 | 120 | -0.202 (0.73) | 0.003 | 140 | -0.005 (0.95) | 0.951 |  |
| ≥70 | 256 | -0.227 (0.90) | <.0001 | 114 | -0.218 (0.80) | 0.004 | 142 | -0.234 (0.98) | 0.005 |  |
| **Executive Functioning** | | | | | | | | | |  |
| All | 941 | -0.219 (0.46) | <.0001 | 400 | -0.185 (0.46) | <.0001 | 541 | -0.244 (0.47) | <.0001 |  |
| 40-49 | 89 | -0.085 (0.48) | 0.0955 | 32 | -0.018 (0.43) | 0.813 | 57 | -0.123 (0.50) | 0.071 |  |
| 50-59 | 227 | -0.134 (0.43) | <.0001 | 100 | -0.110 (0.46) | 0.018 | 127 | -0.153 (0.40) | <.0001 |  |
| 60-69 | 261 | -0.206 (0.43) | <.0001 | 120 | -0.183 (0.39) | <.0001 | 141 | -0.225 (0.46) | <.0001 |  |
| ≥70 | 258 | -0.347 (0.47) | <.0001 | 115 | -0.279 (0.46) | <.0001 | 143 | -0.402 (0.47) | <.0001 |  |
